# Supplementary material for: Finding High-Quality Metal Ion-Centric Regions Across the Worldwide Protein Data Bank
Source: Molecules. 2019 Sep 1;24(17):3179. doi: 10.3390/molecules24173179 (PMC6751499; doi:10.3390/molecules24173179)
Supplement: Supplementary file 1 [file molecules-24-03179-s001.zip › metalloprotein_quality_manuscript_supplemental_material_v3.docx]

**Supplemental Material for Finding high-quality metal ion-centric regions across the worldwide Protein Data Bank**

**Sen Yao^1,2,3^, Hunter N.B. Moseley ^1,2,3,4,5,*^**

^1^ Department of Molecular & Cellular Biochemistry

^2^ Markey Cancer Center

^3^ Resource Center for Stable Isotope Resolved Metabolomics

^4^ Institute for Biomedical Informatics

^5^ Center for Clinical and Translational Science

University of Kentucky, Lexington KY, United States

***** Corresponding Author: hunter.moseley@uky.edu

**Table of Contents**

**Content Page/File**

Supplemental Table 1 – A list of high-quality metal ion list that passes all four criteria in CSV format. S2

Supplemental Figure 1 – Distribution of average absolute electron discrepancy sum within a 3.5 Å radius sphere volume. S3

Supplemental Figure 2 - Distribution of electron discrepancy within 3.5 Å of the metal ion for metal ions within 3 Å of another metal ion. S4

Supplemental Table S1: A list of high-quality metal ion list that passes all four criteria in CSV format.

Filename: TableS1.txt

Column Descriptions:

- pdbid - PDB id
- resolution - resolution of x-ray structure
- chainID - chain ID of the metal ion
- resNum - residue number of the metal ion
- metal - the metal name
- occupancy - the occupancy of the metal
- symmetry - whether or not there are symmetry atoms nearby. -1: no symmetry atom nearby (~5 angstrom range); 0: no symmetry atoms within 3 angstroms; 1: symmetry atoms exist within 3 angstroms sphere.
- totalDensity - the total electron densities within 3 angstroms sphere of the metal, in the unit of e/Å^3^ (same as .ccp4 density map).
- electronNum - the total number of discrepancy electrons after the conversion, in the unit of e.


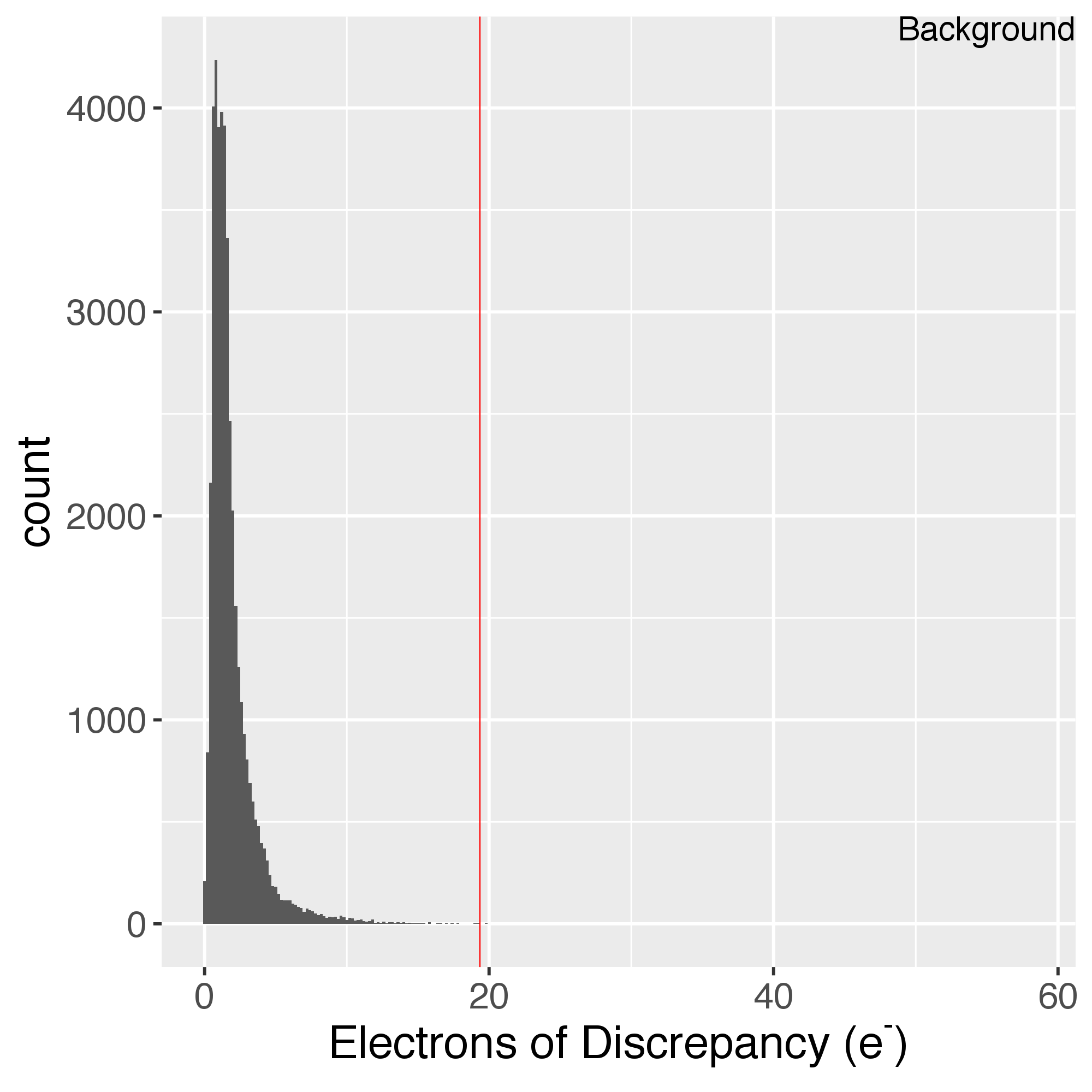


**Figure S1.** Distribution of average absolute discrepancy sum within a 3.5 Å radius sphere volume. The red line indicates the discrepancy cutoff criterion used to filter out low quality metal binding site regions.

**
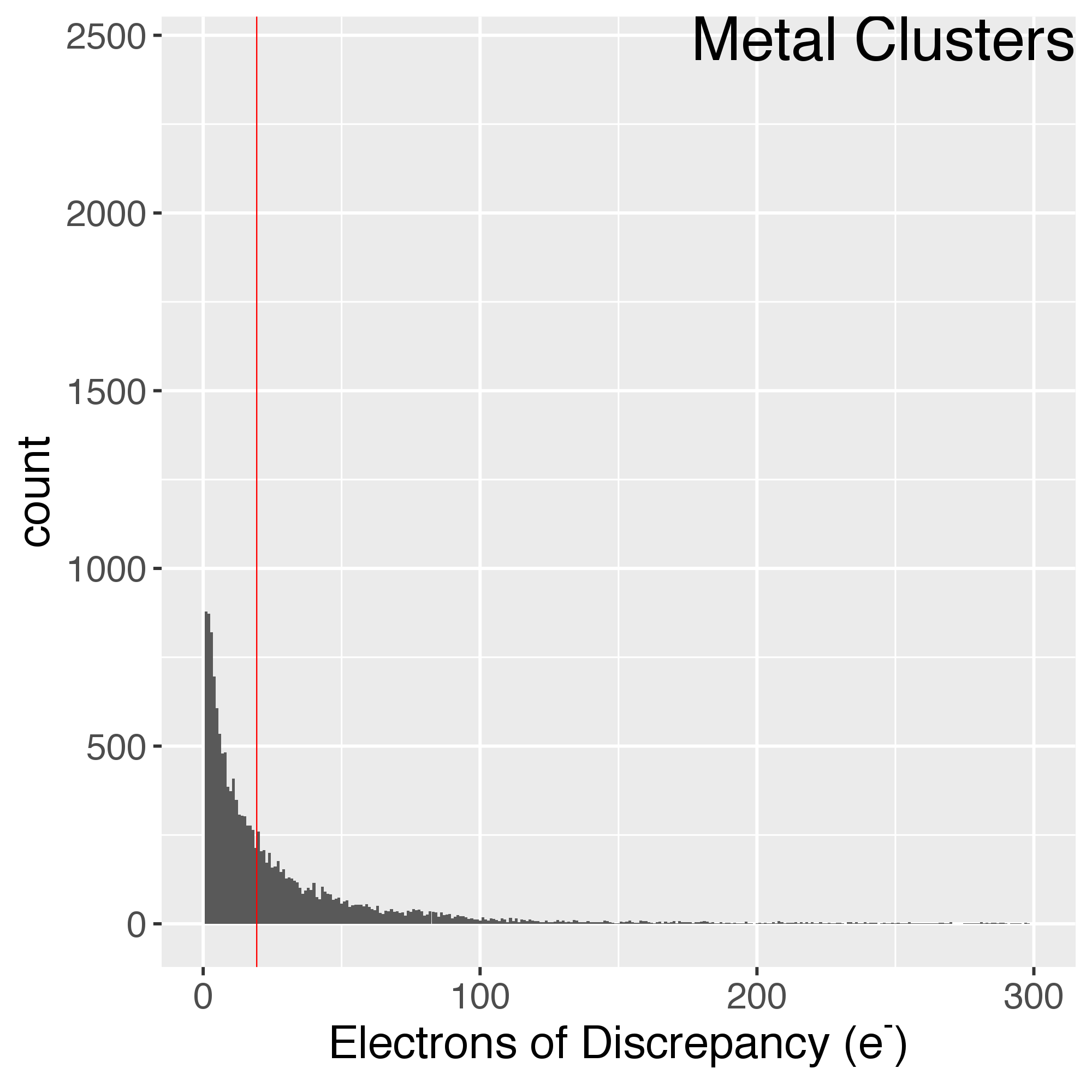
**

**Figure S2.** Distribution of electron discrepancy within 3.5 Å of the metal ion for metal ions within 3 Å of another metal ion. The red line indicates the discrepancy cutoff criterion used to filter out low quality metal binding site regions.
